# Dystrophic Calcinosis Cutis in Systemic Lupus Erythematosus

**DOI:** 10.7759/cureus.8727

**Published:** 2020-06-20

**Authors:** Ikechukwu Achebe, Chimezie Mbachi, Jennifer C Asotibe, Isaac Paintsil

**Affiliations:** 1 Internal Medicine, John H. Stroger, Jr. Hospital of Cook County, Chicago, USA; 2 Medicine, John H. Stroger, Jr. Hospital of Cook County, Chicago, USA

**Keywords:** calcinosis, cutis, sle, dystrophic, lupus, treatment, diffuse, calcium, rheumatology

## Abstract

Calcinosis cutis is a disorder of pathologic calcium deposition in the cutaneous and subcutaneous layers of skin. While common in dermatomyositis and scleroderma, calcinosis cutis less frequently occurs in systemic lupus erythematosus (SLE) and is infrequently described in literature. In this report, we discuss the case of a 36-year-old patient with SLE, presenting with vascular compromise, ulceration, and superimposed infection of her left hand as a consequence of severe calcinosis cutis. This report includes a review of the current literature, and highlights the importance of early detection and intervention in preventing disease complications.

## Introduction

Calcinosis cutis is a disorder characterized by pathologic deposition of insoluble calcium salts in the cutaneous and subcutaneous layers of skin [1]. While common in connective tissue diseases, specifically dermatomyositis, and scleroderma, calcinosis cutis less frequently occurs as a complication of systemic lupus erythematosus (SLE) [2-4]. While the pathophysiology is still poorly understood, calcinosis cutis can be divided into five subtypes depending on mechanism of action: dystrophic, metastatic, idiopathic, iatrogenic, and calciphylaxis [1,5,6].

Dystrophic calcinosis occurs when calcium salts accumulate in areas of ongoing inflammation or previous tissue injury. It is the most common of the five subtypes, and predominates as the subtype most frequently seen in patients with connective tissue disease [6,7]. Complications of calcinosis cutis include chronic pain, cosmetic disfigurement, vascular compromise, ulceration, and superimposed infection. This report details a rare presentation of diffuse dystrophic calcinosis in a patient with SLE, and highlights the importance of early intervention and early detection.

## Case presentation

The patient described is a 36-year-old Hispanic female with medical history of Raynaud’s, SLE (on mycophenolate and hydroxychloroquine), class V lupus nephritis, diffuse calcinosis cutis (with chronic gluteal ulceration), and a recent admission for dry gangrene of the left hand (third digit), who presented to the emergency room with worsening pain and swelling of her left hand, and a new painful ulceration on her right calf.

The patient was recently hospitalized (approximately eight days prior) for the left hand, third-digit ulceration (i.e. dry gangrene). During that admission, workup revealed no ongoing infection. The patient’s extensive calcinosis was found to be causing small artery occlusion in the distal finger which provoked tissue ischemia and death. No surgical intervention was done at that time. 

Since discharge, the patient reported worsening pain in her left hand (third digit, under site of dry gangrene). Her left hand had become swollen, and over the course of seven days, experienced increasing redness around the necrosed fingertip and on the dorsum of her left hand.

The patient followed with rheumatology, dermatology, and renal on an outpatient basis for management of her SLE (with nephritis) and generalized calcinosis cutis. Approximately two months prior, outpatient evaluation revealed progression of her calcinosis cutis. Her treatment regimen included periodic rituximab infusions with topical sodium thiosulfate.

On exam, vitals were stable. Generally, the patient was well appearing. She had scattered subcutaneous and tender nodules present diffusely on her face (chin), chest, upper extremities (elbows, forearms), abdomen, buttocks, and groin. Examination of her left hand revealed dry gangrenous changes of the third digit, moderate hand swelling, and erythematous streaking from the ulceration site in her fingertip to the dorsum of the left hand. A small fluid collection was noted at the base of her ulcer.

Exam of her lower extremities revealed a 3 cm x 3 cm ulcer on the right calf and a 2 cm x 2 cm ulcer on the dorsum of her left foot. Both lesions had surrounding erythema, were warm, and exquisitely tender to touch. No drainage was appreciated.

**Figure 1:**
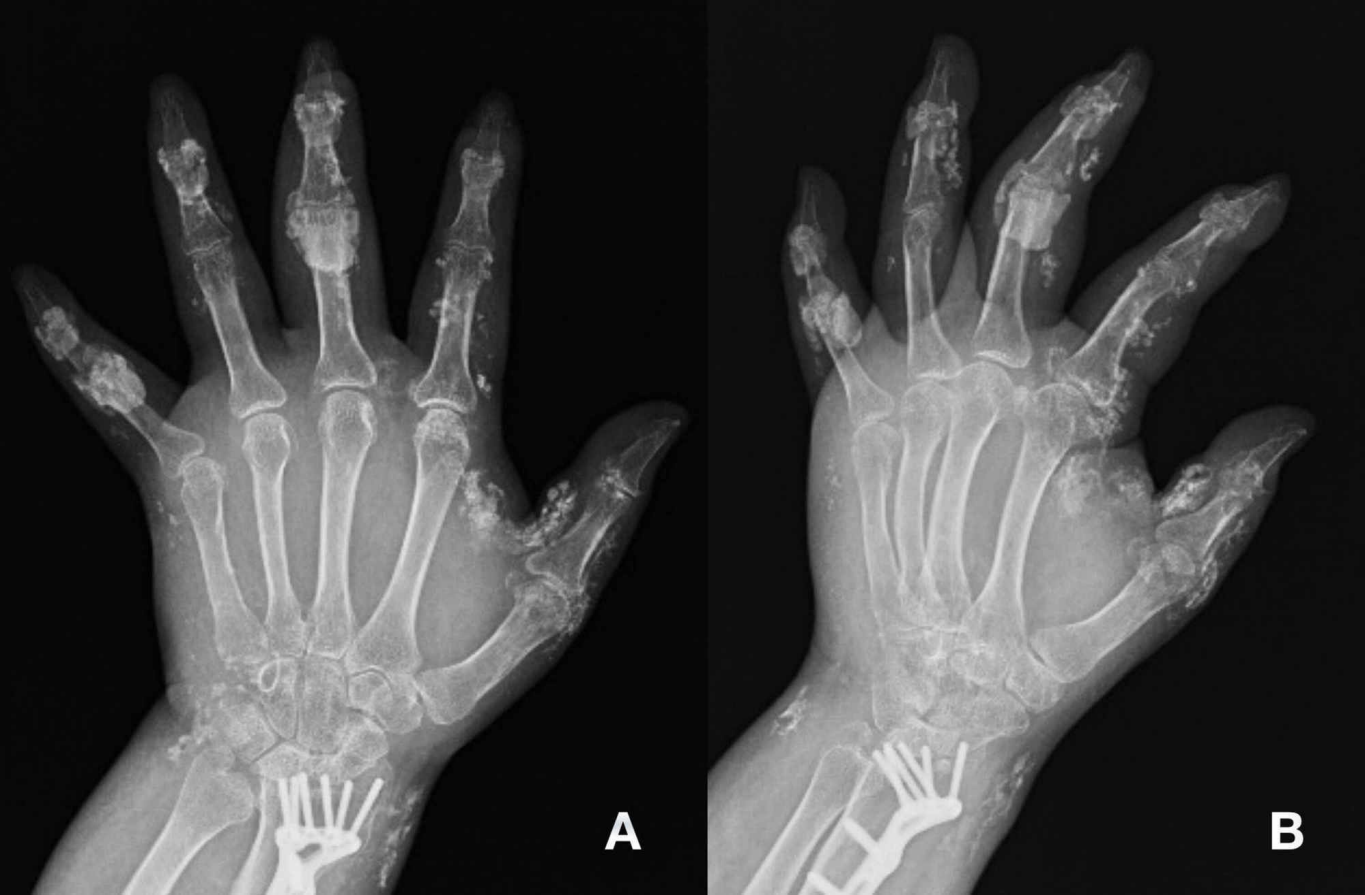
Plain radiograph of the left hand showing extensive diffuse soft tissue calcifications with severe swelling of left forearm, hand, and digits. Images display post-surgical internal fixation that occurred years prior after a motor vehicle accident with left distal radius intra-articular fracture. (A) Anteroposterior view. (B) Oblique view.

Initial workup, which included X-rays of the left hand, revealed severe soft tissue swelling with stable extensive diffuse soft tissue calcifications (Figure 1)

Pertinent laboratory values were as follows: white blood cell count (WBC) = 5.0 K/µL, hemoglobin (Hb) = 10.2 g/dL, platelets = 481 K/µL, C-reactive protein (CRP) was elevated to 14.5 mg/dL. Antinuclear antibody (ANA) was positive; double-stranded DNA (ds-DNA) = 7 (high); anti-centromere antibody was negative; C3 and C4 were within normal limits (wnl) (113 and 24 mg/dL respectively); creatinine = 0.9 (wnl); blood urea nitrogen (BUN) = 53 (high); serum calcium 8.4 (low); phosphorus 5.9 (high).

Blood cultures were taken, and the patient was empirically started on vancomycin in the emergency department. During the course of her admission, the patient would have a bedside incision and drainage of the fluid collection on her left third digit (<1 cc of serous fluid); culture results returned positive for pseudomonas (day 4).

By day 4 of hospital admission, and antibiotic treatment, the patient noted significant improvement in pain and swelling in the left finger (status post [s/p] incision and drainage), and over right calf ulcer. With cultures (left finger) returning positive for pseudomonas, the patient was switched from vancomycin to levofloxacin and was discharge on a regiment of 750 mg QD x 10 days. Although cultures from the patient’s right leg ulcer remained negative, doxycycline at 100 mg Q12 x 7 days was added to her discharge regiment to cover for possible methicillin-resistant Staphylococcus aureus (MRSA) and methicillin-susceptible Staphylococcus aureus (MSSA).

## Discussion

Dystrophic calcinosis cutis is the most common of the calcinosis subtypes, and is the form most commonly seen in patients with associated connective tissue disease. Of the connective tissue diseases, the dystrophic subtype is seen frequently in individuals with limited cutaneous systemic sclerosis (CREST: calcinosis, Raynaud's phenomenon, esophageal dysmotility, sclerodactyly, and telangiectasia), dermatomyositis, and less often in patients with SLE [1-3]. According to the literature, nearly 25% to 40% of individuals with limited cutaneous systemic sclerosis will develop calcinosis within 10 years of disease onset. Similarly, up to 20% of adults with dermatomyositis develop calcinosis cutis. Albeit less frequently, patients with SLE develop calcinosis cutis (up to 17%) [1,3,4,6]. 

End-stage renal disease (ESRD) patients undergoing chronic hemodialysis (HD) also have been shown to have increased rates of calcinosis development. For that reason, it is important to mention that our patient, despite having lupus nephritis, had relatively preserved renal function and was not on HD. Additionally, our patient repeatedly tested negative for anti-centromere antibodies, making competing diagnoses of CREST syndrome or HD-associated calcinosis less likely.

As seen in the described patient, the soft tissue calcifications of calcinosis cutis occur gradually, and can become problematic depending on shape, size, quantity, and degree of functional impairment. Characteristically, lesions occur in the upper extremities (elbows, forearms, and interphalangeal joints); however, patients with SLE often develop lesions in the buttocks (seen in this patient), peri-auricular area, and underneath cutaneous lupus lesions [1,3].

Calcinosis cutis is notoriously difficult to treat. Typically, it occurs 21.5 years after SLE is diagnosed and thus, prompt identification and early intervention are crucial to preventing disease propagation [1]. This report sheds light on the nature, and frequency of complications that arise as a consequence of late-stage disease. Because a major complication of calcinosis cutis is vascular compromise leading to tissue ischemia and death, patients should first be instructed to reduce risk factors that further compromise peripheral perfusion (smoking, stress, cold exposure) [1,4]. Additionally, patients with connective tissue disease can slow progression of dystrophic calcinosis by adequately suppressing the activity of their associated auto immune disease, reducing the amount of damaged tissue which serves as a nidus for calcium deposition. Depending on disease severity, pharmacologic and or surgical intervention should be considered.

To date, there are approximately 45 documented cases of cutaneous cutis occurring in SLE, and no randomized controlled trials elevating the efficacy of existing and emerging therapies. While not universally efficacious, oral therapies including diltiazem, colchicine, and minocycline are often used as initial agents in patients with generalized, dystrophic calcinosis given their relative safety [1,2,4,5]. Diltiazem, when used at high doses (2-4 mg/kg/day), inhibits intracellular calcium absorption in damaged tissue [4]. Minocycline (50-100 mg/day) and colchicine function in the anti-inflammatory cascade reducing tissue destruction and ulceration [1,2,4]. Surgical excision is a common treatment modality proven to be beneficial; especially in regards to the treatment of discrete and localized lesions [1,4].

Other agents that have shown to be efficacious include warfarin, bisphosphonates, ceftriaxone, aluminum hydroxide, probenecid, sodium thiosulfate (topical, IV), intralesional corticosteroids, lithotripsy, and rituximab. Factors such as comorbidities, side effect profile, patient tolerance, and response to therapy should be used to help guide decision making [2,4,8].

## Conclusions

Calcinosis cutis is a disorder of pathologic calcium deposition in cutaneous and subcutaneous layers of skin. Although rare, patients with SLE present most commonly with the dystrophic subtype of calcinosis. Early identification and intervention are crucial to preventing disease propagation. While the literature is limited, efficacious treatments exist in the form of oral therapy, infusions, topical creams, and surgical intervention. These therapies, when used early and in combination with risk factor reduction, can prevent chronic pain, cosmetic disfigurement, vascular compromise, ulceration, and superimposed infections that patients suffer in advanced and diffuse disease.

## References

[1] Balin SJ, Wetter DA, Andersen LK, Davis MD (2012). Calcinosis cutis occurring in association with autoimmune connective tissue disease: the Mayo Clinic experience with 78 patients, 1996-2009. Arch Dermatol.

[2] Dima A, Balanescu P, Baicus C (2014). Pharmacological treatment in calcinosis cutis associated with connective-tissue diseases. Rom J Intern Med.

[3] Huang HL, Wu WT, Ou TT (2014). Extensive calcinosis cutis universalis in a patient with systemic lupus erythematosus: 10-year treatment experience. Kaohsiung J Med Sci.

[4] Le C, Bedocs PM (2020). Calcinosis Cutis. Updated.

[5] Lopez AT, Grossman ME (2017). Facial calcinosis cutis in a patient with systemic lupus erythematosus: a case report of tissue injury owing to photosensitivity as the cause of dystrophic calcification. JAAD Case Rep.

[6] Reiter N, El-shabrawi L, Leinweber B, Berghold A, Aberer E (2011). Calcinosis cutis: part I. Diagnostic pathway. J Am Acad Dermatol.

[7] Walsh JS, Fairley JA (1995). Calcifying disorders of the skin. J Am Acad Dermatol.

[8] Wolf EK, Smidt AC, Laumann AE (2008). Topical sodium thiosulfate therapy for leg ulcers with dystrophic calcification. Arch Dermatol.

